# Expression of MYCN in Multipotent Sympathoadrenal Progenitors Induces Proliferation and Neural Differentiation, but Is Not Sufficient for Tumorigenesis

**DOI:** 10.1371/journal.pone.0133897

**Published:** 2015-07-29

**Authors:** Bret C. Mobley, Minjae Kwon, Bradley R. Kraemer, F. Edward Hickman, Jingbo Qiao, Dai H. Chung, Bruce D. Carter

**Affiliations:** 1 Department of Pathology, Microbiology, and Immunology, Division of Neuropathology, Vanderbilt University Medical Center, Nashville, Tennessee, the United States of America; 2 Department of Biochemistry, Vanderbilt University Medical Center, Nashville, Tennessee, the United States of America; 3 Vanderbilt Brain Institute, Vanderbilt University Medical Center, Nashville, Tennessee, the United States of America; 4 Department of Pediatric Surgery, Vanderbilt University Medical Center; Nashville, Tennessee, the United States of America; University of Montréal and Hôpital Maisonneuve-Rosemont, CANADA

## Abstract

Neuroblastoma is a pediatric malignancy of the sympathetic ganglia and adrenal glands, hypothesized to originate from progenitors of the developing sympathetic nervous system. Amplification of the *MYCN* oncogene is a genetic marker of risk in this disease. Understanding the impact of oncogene expression on sympathoadrenal progenitor development may improve our knowledge of neuroblastoma initiation and progression. We isolated sympathoadrenal progenitor cells from the postnatal murine adrenal gland by sphere culture and found them to be multipotent, generating differentiated colonies of neurons, Schwann cells, and myofibroblasts. MYCN overexpression in spheres promoted commitment to the neural lineage, evidenced by an increased frequency of neuron-containing colonies. MYCN promoted proliferation of both sympathoadrenal progenitor spheres and differentiated neurons derived from these spheres, but there was also an increase in apoptosis. The proliferation, apoptosis, and neural lineage commitment induced by MYCN are tumor-like characteristics and thereby support the hypothesis that multipotent adrenal medullary progenitor cells are cells of origin for neuroblastoma. We find, however, that MYCN overexpression is not sufficient for these cells to form tumors in nude mice, suggesting that additional transforming mutations are necessary for tumorigenesis.

## Introduction

Neuroblastoma is the most common cancer in infants and the most common extracranial tumor of childhood [1,2]. Neuroblastomas arise from the developing sympathetic nervous system, with half of tumors originating in the adrenal medulla, and the remainder arising in paraspinal sympathetic ganglia of the chest, abdomen, pelvis, or neck [1,3]. While neuroblastoma patient outcomes have improved over the last several decades, a significant proportion of patients do not survive their disease; the ten-year survival is 70%, and for patients with “high risk” clinical, histologic, and molecular features the ten-year survival is less than 50% [4,5]. In addition, current treatment regimens cause long-term complications including hearing impairment, endocrine disturbances, and orthopedic problems in a large percentage of survivors [6–8]. The anatomical sites at which neuroblastomas arise and their gene expression profiles suggest that these tumors arise from sympathoadrenal progenitors [9,10]. It has been hypothesized that neuroblastoma and other embyronal tumors arise as a result of impaired differentiation, driven by tumor initiating cells that are unable to undergo terminal differentiation. Studying the development of sympathoadrenal progenitors and the changes in behavior they show in the context of oncogene expression may therefore improve our understanding of disease initiation and progression.

*MYCN* is a member of the *MYC* oncogene family originally identified in human neuroblastoma [11], and subsequently found to be expressed in the newborn murine adrenal gland [12]. Soon after its discovery, amplification of *MYCN* was found to correlate with poor prognosis in neuroblastoma patients [13], and amplification is routinely assayed in the clinical setting to stratify risk. A strong link to a neural crest-derived cell of origin for neuroblastoma was established when mice overexpressing MYCN in neural crest cells under the tyrosine hydroxylase promoter were shown to develop neuroblastoma-like tumors, specifically in the paraspinal sympathetic ganglia [14,15]. Similarly, MYCN expression was shown to drive tumor development from a neural crest cell line [16]. It has also been shown that expression of MYCN can induce tumor formation in the zebrafish interrenal gland, the equivalent of the mammalian adrenal gland [17]. Despite these advances, very little is known about the role of MYCN in the early steps of neuroblastoma initiation.

It has recently been shown that multipotent sympathoadrenal progenitor cells (SAPs) can be isolated from the adrenal gland. Chung and colleagues first demonstrated the presence of sphere-forming progenitor cells in the adult bovine adrenal medulla, capable of producing functionally mature neurons in the presence of NGF and chromaffin cells in the presence of dexamethasone [18]. The same group went on to describe progenitor cells in the adult human adrenal gland [19]. Most recently, SAPs were isolated from the adrenal glands of postnatal mice; these cells grew as spheres in non-adherent conditions and expressed the sympathoadrenal progenitor marker *Phox2b* together with the neural crest stem cell associated genes *Bmi1*, *Sox10*, and *Mycn* [20].

While the adrenal gland is a frequent site of neuroblastoma origin, the impact of MYCN expression on multipotent mammalian SAPs has not been described. We isolated SAPs from the postnatal murine adrenal gland by clonal sphere culture and found that they are multipotent, capable of generating the well characterized neural crest derivatives: neurons, Schwann cells, and myofibroblasts [21,22]. MYCN overexpression in these cells markedly shifted their differentiation toward the neural lineage, compatible with the neural histologic phenotype observed in neuroblastoma. We also show that MYCN enhanced the proliferation of murine SAP spheres and adherent sphere-derived sympathetic neurons, while imparting increased sphere-forming capacity. Nevertheless, MYCN overexpressing SAPs were not able to form tumors in nude mice.

## Materials and Methods

### Ethics Statement

This study was carried out in strict accordance with the recommendations in the Guide for the Care and Use of Laboratory Animals of the National Institutes of Health. The protocol was approved by the Vanderbilt Institutional Animal Care and Use Committee. All efforts were made to minimize suffering.

### Culture of Adrenal Gland Cells

Adrenal glands were harvested from postnatal day 0/1 C57BL/6 mice, dissociated, and grown as spheres using neural crest stem cell procedures modified from Morrison and colleagues [21,23,24]. Freshly harvested tissue was dissociated for 30 minutes at 37°C with 0.15% collagenase (Sigma), 0.06% trypsin (Worthington), and 150 units/ml deoxyribonuclease (DNAse)(Sigma) in HBSS (GIBCO) plus 0.53 mM EDTA (Sigma). The digest was quenched in staining medium including L15 (GIBCO) with 1 mg/ml BSA (Sigma), 10 mM HEPES (GIBCO), 0.53 mM EDTA, and 15 units/ml DNAse. Cells were initially plated on 35mm TC dishes (Greiner Bio-One), and at two hours non-adherent cells within the supernatant were transferred to ultra-low attachment surface plates (Costar). Cells were grown at a clonal density of 1 cell per microliter (S1 Fig) in self-renewal medium including a 5:3 mixture of DMEM-low glucose:neurobasal medium (Invitrogen) supplemented with 20 ng/ml basic fibroblast growth factor (bFGF)(StemCell Technologies), 1% N2 (Invitrogen), 2% B27 (Invitrogen), 50 μM β-mercaptoethanol (Sigma), 117nM retinoic acid (Sigma), 15% chick embryo extract (CEE), and 1% penicillin/streptomycin (Gibco).

Spheres were grown for five days and differentiated for colony composition assessment by transfer to differentiation medium (10ng/ml bFGF and 1% CEE) in 48-well tissue culture dishes sequentially coated with 0.15 mg/ml poly-d-lysine (MP Biomedicals) and 0.2 mg/ml human fibronectin (Biomedical Technologies). Colonies were maintained in differentiation medium for 48 hours prior to fixation with 4% paraformaldehyde (Sigma) for immunohistochemical analysis. For secondary and tertiary sphere formation assays, primary spheres were dissociated in a mixture of 10 units/ml papain (Worthington), 470 units/ml type IV collagenase (Worthington), 600 units/ml DNAse, and 40% Accutase (Millipore) per volume with frequent agitation. The digest was quenched in staining medium, the dissociated single cells were pelleted, and an additional quench step with ovomucoid protease inhibitor (Worthington) was performed. Cells were then plated at a clonal density of 1 cell per microliter of self-renewal medium (S1 Fig). All cultures were maintained at 37°C in gas-tight chambers flushed with 1% O_2_/6% CO_2_/balance N_2_ to achieve a physiologic oxygen concentration of 5% [23].

### Quantitative Real Time PCR (qRT-PCR)

TRIzol reagent (Life Technologies) was used to isolate total RNA from primary spheres on day 5 of culture or from postnatal day 3 superior cervical ganglion (SCG) or adrenal gland. cDNA was synthesized using SuperScript III reverse transcriptase (Life Technologies). qPCR reactions were prepared in 10 ul volumes in triplicate using SsoAdvanced SYBR Green Supermix (Bio-Rad) following the manufacturer’s instructions. qRT-PCR was performed using the CFX96 Real-Time PCR Detection System (primer sequences listed in S1 Table). Cycling conditions were 95°C for 10 min, followed by 40 cycles with 95°C for 15 s, 55°C for 30 s, and 72°C for 30 s. After the final cycle, melting curve analysis was performed to confirm correct product amplification. Tbp1 was used as the endogenous reference gene, where the fold difference between the gene of interest and Tbp1 = 2^(mean Ct Tbp1—mean Ct gene of interest)^.

### Lentiviral construct and infection

The wild type mouse *Mycn* gene including an N-terminal Flag tag, kindly provided by Dr. Anna M. Kenney, was cloned into the pHIV-Zsgreen lentiviral vector. 293T cells were transfected with pHIV-Zsgreen vector with or without the *Mycn* gene, the packaging plasmid psPAX2, and the envelope plasmid pCI-VSVG. Viral supernatant was concentrated by ultracentrifugation and incubated overnight with sphere-forming cells isolated by differential plating. A functional virus titer of 7.55 x 10^7^ transducing units per ml was determined by 293T cell infection using the formula: titer = {F x Co/V} x D where F is the frequency of GFP-positive cells determined by flow cytometry, Co is the number of target cells exposed to virus, V is the volume of the inoculum, and D is the virus dilution factor [25]. Adrenal progenitor cells isolated by differential plating were infected overnight with a 1:3 dilution of virus in self-renewal medium using 96-well ultra-low attachment surface plates (Costar), and transferred to 24-well ultra-low attachment plates (Costar) the following morning for primary sphere formation. Primary spheres were grown for 4 days to allow oncogene expression prior to dissociation for differentiation, proliferation, apoptosis, or tumor formation assay.

### Western Blot Analysis

Whole spheres were lysed with RIPA buffer (50 mM Tris HCl (pH 8.0), 150 mM, NaCl, 2 mM EDTA, 1% sodium orthovanadate, 1% Triton X-100, 0.5% deoxycholate, 0.1% sodium dodecyl sulfate) and 40ug of cell extract was separated using SDS–10% PAGE and transferred to a Nitrocellulose membrane (Protran, Whatman). After blocking with 5% milk powder, the membrane was incubated with anti-N-myc antibody (Cell Signaling #9405, 1:1000) or anti-alpha-tubulin antibody (1:5000, Calbiochem Cat# CP06), followed by incubation with anti-rabbit IgG HRP (Cell Signaling) or anti-mouse IgG HRP, respectively. The membranes were rinsed and visualized with ECL Western Blotting Substrate (Pierce).

### Proliferation Assay, Apoptosis, and Immunohistochemistry

To assess the proliferation rate of cells in secondary spheres, 10 μM EdU (Click-iT imaging kit, Invitrogen) was added to the self-renewal medium at day 3 of growth for a 24-hour interval. Spheres were dissociated to single cells, plated on poly-d-lysine and fibronectin coated dishes in self renewal medium, and fixed with 4% paraformaldehyde following attachment at 1.5 hours. EdU staining was performed per manufacturer’s instructions. Apoptosis was measured by TUNEL labeling (ApopTag Fluorescein Direct In Situ Apoptosis Detection Kit, Millipore) in the same samples. To assess the proliferation rate of adherent cells, the spheres were dissociated and the cells plated on poly-d-lysine and fibronectin coated dishes in differentiation medium. After 24 hours in culture, EdU was added to the medium for a 24-hour period after which time the cells were fixed and stained as above. Primary antibodies used to assess differentiation by immunocytochemistry included those against neuronal class III β-tubulin (TUJ1)(Covance, MMS-435P, 1:400), S100 (Immunostar, 22520, 1:1), smooth muscle actin (Sigma, A5228, 1:200), and tyrosine hydroxylase (Millipore, AB152, 1:400) following block/permeabilization with 10% goat serum and 0.1% Triton X-100. Alexa Fluor 488 or 546-conjugated secondary antibodies (Invitrogen, 1:1000) were used. Cells were counterstained with 2.5 μg/ml 4’,6-diamino-2-phenylindole dihydrochloride (DAPI) to visualize the nuclei. Images were acquired on a Leica DM IRB inverted microscope. For colony composition analysis, S100 non-reactive/TuJ1 non-reactive cells with large nuclei and flat morphology were counted as myofibroblasts, as cells with these cytologic features routinely demonstrated SMA-reactivity. Primary antibodies used to examine spheres included nestin (Millipore, MAB 353, 1:200), tyrosine hydroxylase (Millipore, AB152, 1:400), and SOX10 (Cell Marque, PA0813). On day 5 of culture, spheres were fixed with 4% PFA for 10 minutes at room temperature, washed with PBS, embedded in OCT, and sectioned at 10 μm intervals. Block/permeabilization with 5% BSA plus 0.2% Triton X-100 or 10% goat serum plus 0.3% Triton X-100 was performed prior to staining for nestin and TH, respectively. These were followed by an Alexa Fluor 488-conjugated secondary antibody (Invitrogen, 1:1000) and DAPI counterstain. Images were acquired on a Zeiss LSM 710 META inverted confocal microscope. For SOX10 immunoperoxidase staining, fixed spheres were pelleted, processed in Histogel (Richard-Allan) on a tissue processor, embedded in paraffin, and sectioned at 5 μm. Slides were deparaffinized and heat induced antigen retrieval was performed on the Leica Bond Max IHC stainer using Epitope Retrieval 2 solution for 10 minutes. The sections were incubated with anti-SOX10 for one hour and the Bond Refine Polymer detection system was used for visualization. Sections were visualized using an Olympus BX40 light microscope.

### Tumor Formation Assay

Male athymic nude mice (4–6 weeks old) were maintained as previously described [26]. All studies were approved by the Institutional Animal Care and Use Committee at Vanderbilt University and were conducted in accordance with NIH guidelines. 5 x 10^4^ BE (2)-C cells or MYCN-overexpressing adrenal progenitor cells were resuspended in 100 μl of HBSS including 30% Matrigel (BD Biosciences) and injected subcutaneously into the right flank using a 26-gauge needle (n = 7 per group). Tumor growth was assessed by measuring the two greatest perpendicular tumor dimensions with vernier calipers (Mitutoyo) and body weights were recorded weekly.

### Statistical Analyses

GraphPad Prism 5 software was used for statistical analysis. All statistics were mean ± s.e.m. and p-values were calculated by one-way ANOVA or with Student’s t-test.

## Results

### MYCN promotes neural lineage commitment in multipotent sympathoadrenal progenitors

**Fig 1:**
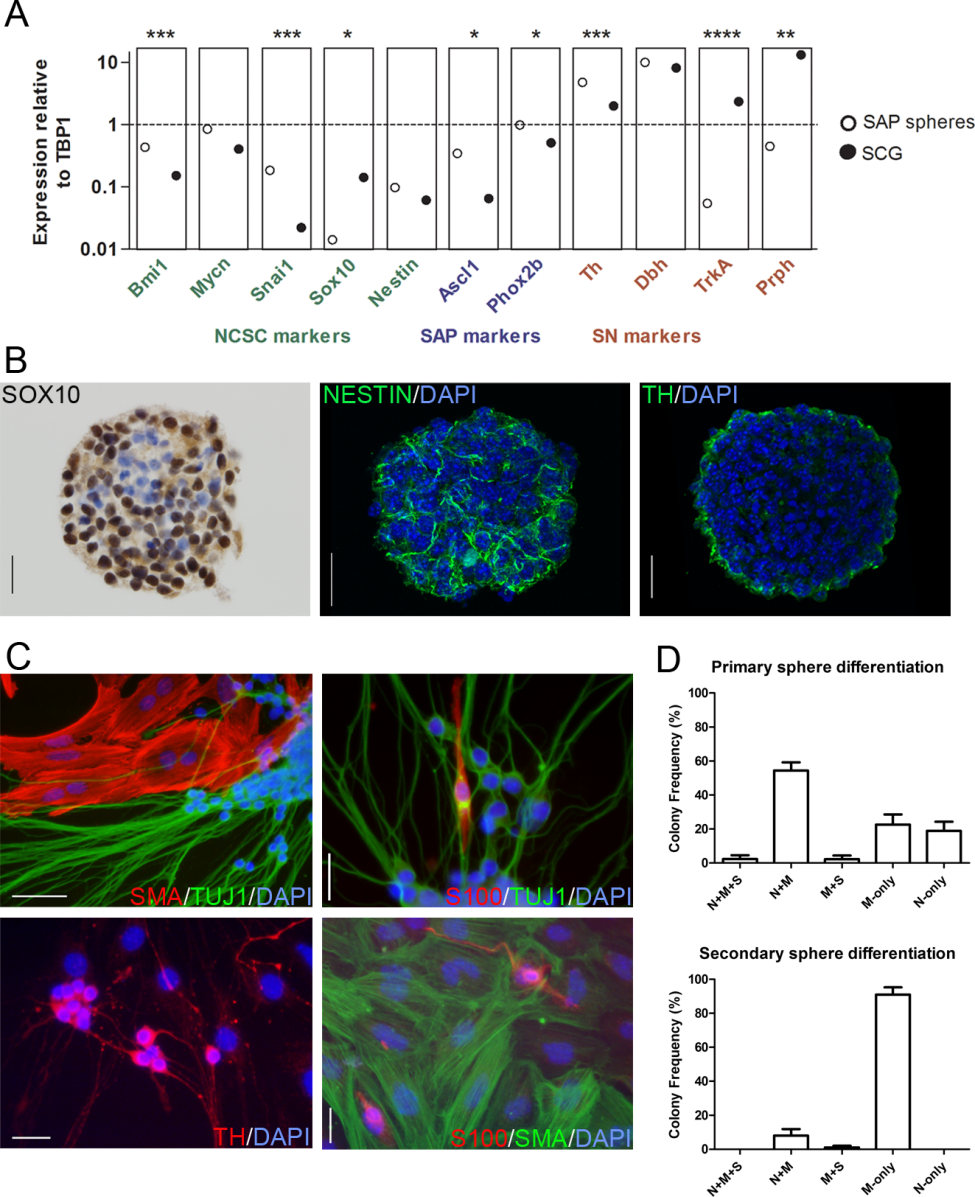
Sympathoadrenal Progenitor Sphere Characterization and Differentiation. (A) Spheres grown from postnatal mouse adrenal glands cells express markers of neural crest stem cells (NCSCs), sympathoadrenal progenitors (SAPs), and sympathetic neurons (SNs). mRNA levels were analyzed by qRT-PCR and are shown in relation to TATA-Box binding protein-1 (TBP1). Expression data from postnatal mouse superior cervical ganglia (SCG) are included for comparison. Data are the mean values from four experiments run in triplicate. *p<0.05,**p<0.01,***p<0.001,****p < .0001, unpaired Student’s t-test. (B) The majority of sphere cells show nuclear SOX10 reactivity by immunoperoxidase labeling. Cytoplasmic nestin expression with linear morphology was observed by immunofluorescence microscopy in spheres. TH expression was prominent at the periphery of spheres. Representative images from 3 independent experiments. Scale bars equal 20 microns. (C) Sphere differentiation was induced by transfer to poly-d-lysine and fibronectin-coated plates in medium with low CEE content. Immunofluorescent images show the three cell types observed in differentiated spheres: TuJ1/TH-reactive neurons, SMA-reactive myofibroblasts, and S100-reactive Schwann cells. Scale bars 50 μM upper left panel and 25 μM all other panels. (D) A shift in differentiation potential was observed from primary spheres, grown directly from adrenal gland cells by differential plating, to secondary spheres, the products of dissociated primary spheres. Differentiated secondary spheres showed a reduced frequency of bipotent and neuron-containing colonies, and were more likely to show myofibroblast-only colonies (primary spheres n = 3, secondary spheres n = 4). Primary and secondary spheres were grown at clonal density.

Recent studies have shown that multipotent sympathoadrenal progenitor cells (SAPs) can be isolated from the adrenal gland by differential plating and grown as spheres [18–20]. We employed this strategy to study the impact of *Mycn*, the murine homolog of *MYCN*, on neural progenitor cell differentiation and proliferation. Postnatal mouse adrenal progenitors were grown as spheres in chick embryo extract (CEE)-containing self-renewal medium at physiologic oxygen levels [23]. Cells were plated at a density of one cell per μL, a clonal density at which spheres formed would be derived from single cells (S1 Fig). Quantitative PCR was used to examine gene expression in spheres, and expression levels were compared to those found in postnatal superior cervical ganglia (SCG), a sympathoadrenal tissue containing neurons and Schwann cells. Spheres expressed genes characteristic of neural crest stem cells (NCSCs) including Bmi1, Mycn, Snai1, Sox10, and Nestin (Fig 1A). Bmi1 and Snai1 were expressed more highly by spheres than by SCG tissue. Mycn and Nestin showed greater expression on average in spheres, but the differences did not reach statistical significance. Sox10 expression was higher in sympathetic ganglia, where it is expressed by mature glial cells. Expression of the SAP genes Ascl1 and Phox2b was also greater in spheres, while the sympathetic neuron (SN) markers TrkA and Peripherin were more highly expressed by SCG. Tyrosine hydroxylase and dopamine β hydroxylase were expressed highly by the two tissue types, indicating that adrenal-derived spheres express markers common to neural crest progenitors and sympathetic neurons. Quantitative PCR for the adrenal cortical markers Sf-1, Cyp11a1, and Cyp11b2 revealed no significant expression in spheres relative to postnatal adrenal gland control (data not shown). Immunohistochemical studies of primary spheres demonstrated expression of the NCSC proteins SOX10 and nestin (Fig 1B), with expression of tyrosine hydroxylase at the periphery of spheres.

Spheres were then transferred to differentiation medium, characterized by low CEE levels and including retinoic acid, on poly-d-lysine and fibronectin-coated plates to determine differentiation potential. Primary adrenal spheres formed colonies containing both TuJ1-immunoreactive neurons (N) and smooth muscle actin (SMA)-reactive myofibroblasts (M), with rare tri-lineage colonies including S100-positive Schwann cells (S) with spindled morphology (Fig 1C and 1D). Colonies demonstrating only neurons (N-only) or myofibroblasts (M-only) were seen as well. Neurons showed tyrosine hydroxylase (TH) reactivity indicative of sympathoadrenal differentiation. Primary spheres were then dissociated and plated at clonal density to generate secondary spheres. Limited self-renewal was observed: bipotent N+M colonies were seen, although these were reduced in number relative to the primary spheres (Fig 1D). Neuron-containing colonies were decreased in frequency overall as well, with no N-only colonies observed.

**Fig 2:**
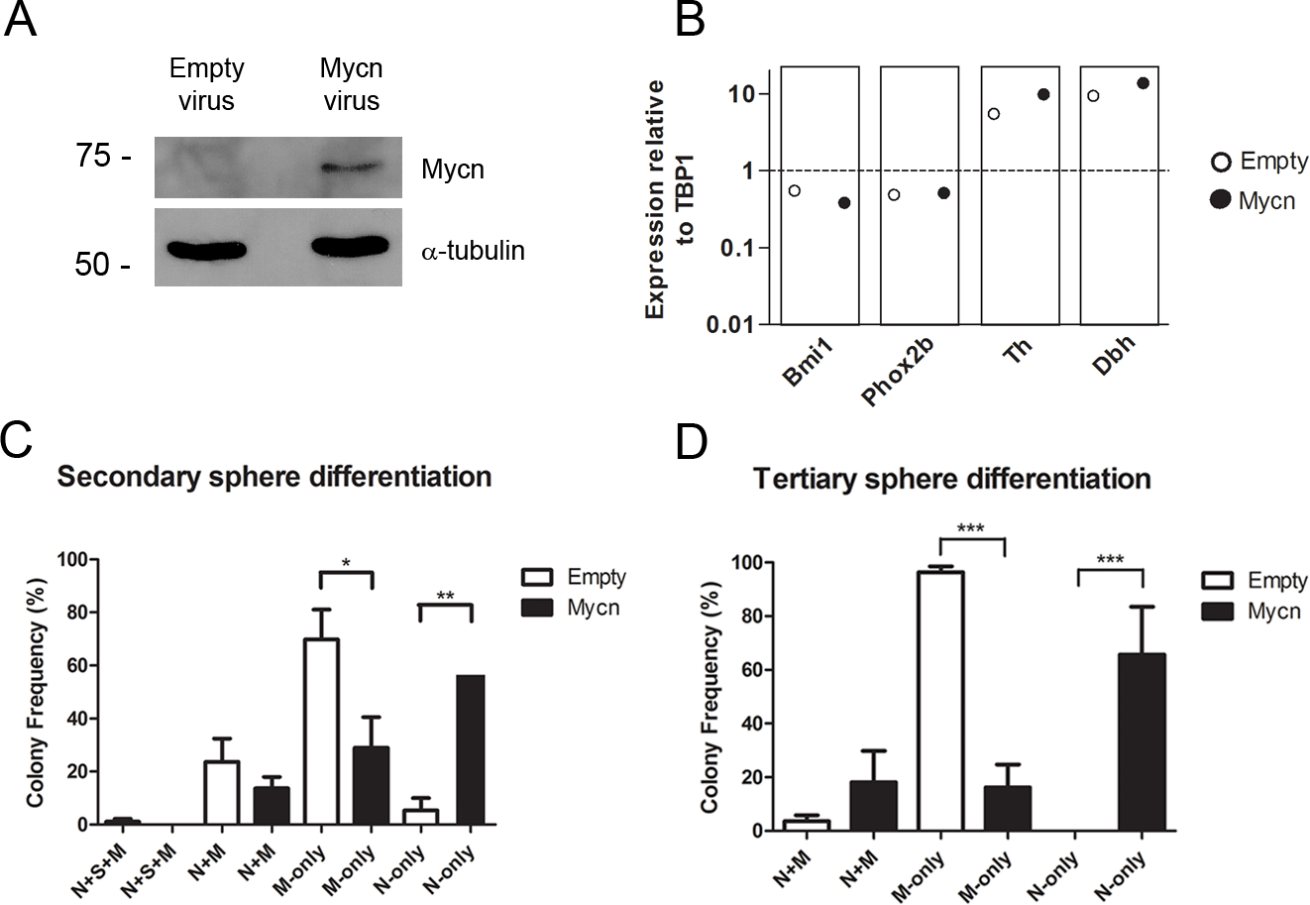
MYCN Overexpression Induces Neuronal Lineage Commitment. (A) Adrenal progenitor cells isolated by differential plating were infected with empty or *Mycn* lentivirus, and 3 to 4 days following infection the resulting primary spheres were analyzed for MYCN expression by western blot. (B) Spheres infected with *Mycn* lentivirus showed lower mean levels of Bmi1 and increased Dbh and Th levels, though differences did not reach statistical significance (unpaired Student’s t-test). Data are the mean values from four experiments run in triplicate. mRNA levels from primary spheres were analyzed by qRT-PCR and are shown in relation to TATA-Box binding protein-1 (TBP1). (C) MYCN expression induced neuronal lineage committment in differentiated secondary spheres with a markedly increased frequency of N-only colonies and a substantial reduction of M-only colonies (n = 5). (D) Neural lineage induction by MYCN was maintained in the subsequent passage to tertiary spheres: N-only colonies were observed with MYCN overexpression but were absent from differentiated control spheres (n = 3). Statistical analysis was performed using one-way analysis of variance with Tukey’s multiple comparison test. *p<0.05,**p<0.01, ***p < .001.

To explore the effects of MYCN overexpression on sympathoadrenal progenitor lineage committment, we delivered the oncogene to freshly harvested cells by lentiviral transduction. Primary spheres were allowed to form, and western blot confirmed overexpression of the MYCN protein by spheres (Fig 2A). Quantitative PCR showed a lower mean level of the NCSC marker *Bmi1* in the setting of MYCN expression, and increased levels of the sympathetic neural lineage markers *Dbh* and *Th* (Fig 2B). These differences, however, did not reach statistical significance. Primary spheres were then dissociated for secondary sphere growth at a density of one cell per microliter, and the resulting secondary spheres were differentiated to assess the impact of MYCN expression on colony composition. Interestingly, with MYCN expression, differentiated colonies were significantly more likely to contain neurons: N-only colonies were observed at a markedly increased frequency with a substantial reduction of M-only colonies (Fig 2C). Neural lineage induction by MYCN was maintained in the subsequent passage to tertiary spheres (Fig 2D), where N-only colonies were absent from controls but constituted the majority of MYCN overexpressing colonies. Control spheres were again more likely to form M-only colonies in this passage. While MYCN expression in development and neoplasia inhibits neural differentiation to maintain cells in a precursor state [27–30], our results indicate that MYCN strongly promotes the neural lineage in multipotent SAPs. This finding is consistent with the neural lineage commitment seen in neuroblastomas.

### MYCN induces proliferation of sympathoadrenal progenitors and neurons

**Fig 3:**
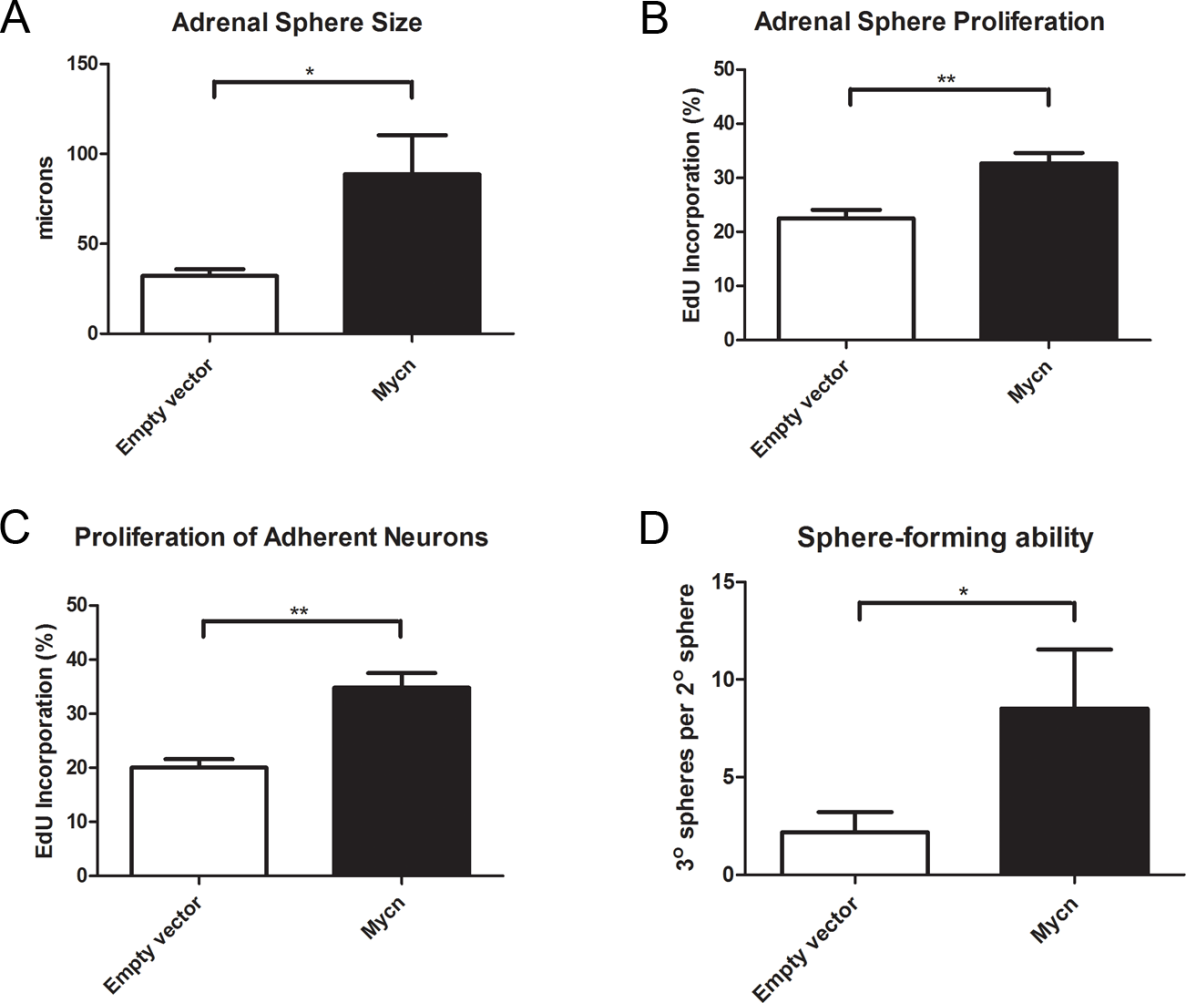
MYCN Enhances Proliferation and Sphere-Forming Ability. (A) MYCN expression resulted in increased sphere size, compatible with a pro-proliferative effect on sympathoadrenal progenitor cells. Adrenal progenitor cells were infected with empty or *Mycn* lentivirus, primary spheres were grown for 4 days to allow expression of the oncogene, and then primary spheres were dissociated and plated at clonal density for secondary sphere formation. The diameters of the resulting spheres were measured 5 days later. (n = 4, p < .05, Student’s t-test). (B) MYCN induced proliferation of sphere cells as evidenced by increased EdU incorporation. 10μM EdU was added to the self-renewal medium of MYCN expressing or control secondary spheres at day 3 of growth for a 24-hour interval. Sphere cells were dissociated, plated on coated dishes in self-renewal medium, and fixed and stained following attachment (n = 4, p < .01, Student’s t-test). (C) MYCN induced proliferation of adherent, process-bearing, TuJ1-reactive neurons. Primary adrenal sphere cells were dissociated 4 days after infection with either empty or *Mycn* lentivirus, and plated on poly-d-lysine and fibronectin coated dishes in differentiation medium. After 1 day in culture, EdU was added to the medium for a 24-hour period after which time the cells were fixed and stained (n = 5, p < .01, Student’s t-test). (D) MYCN enhanced sphere-forming ability with greater numbers of tertiary spheres formed per secondary sphere dissociated. Secondary spheres expressing the *Mycn* gene or a control construct were counted, dissociated, and plated at a density of one cell per microliter. After 7 days of growth the number of tertiary spheres was counted and the ratio of tertiary spheres formed to secondary spheres dissociated was determined (n = 6, p < .05, paired Student’s t-test).

MYCN is required for the normal expansion of neural precursors during development of the mammalian forebrain and hindbrain [30]. Correspondingly, MYCN upregulation promotes proliferation in many nervous system tumors [31–33] and amplification of *MYCN* correlates with poor prognosis in neuroblastoma patients [13]. To better understand the role of MYCN in oncogenesis, proliferation assays were performed on SAPs, the likely cells of origin for neuroblastoma. Progenitor cells were isolated from freshly harvested adrenal cells and exposed to MYCN expressing or control lentivirus. Primary spheres were grown for 3–4 days to allow expression of the oncogene, and then dissociated for secondary sphere formation. MYCN overexpressing spheres grew roughly twice as large as control spheres (Fig 3A), suggesting enhanced proliferation. When proliferation was measured directly by EdU incorporation, MYCN overexpressing sphere cells showed a significantly higher proliferation rate (Fig 3B).

Sympathoadrenal neuronal precursors retain the ability to proliferate after the acquisition of neuronal properties [34–37], and a recent study showed that precursors temporarily exit the cell cycle upon expression of TuJ1 and TH with subsequent cycle re-entry before terminally exiting [38]. To learn the impact of MYCN overexpression on adherent, process-bearing neurons, spheres infected with control or MYCN expressing lentivirus were dissociated to single cells and plated in differentiation medium on poly-d-lysine and fibronectin-coated plates. EdU was added to the medium 24 hours after plating, by which point neuronal cells had extended processes, and the cells were fixed 24 hours later. We observed that TuJ1-reactive neurons derived from MYCN overexpressing sphere cells were significantly more proliferative than control infected neurons (Fig 3C), demonstrating that MYCN induces proliferation not only of sympathoadrenal progenitors grown as spheres, but also of neurons grown adherently.

To learn the impact of MYCN expression on sphere-forming ability by SAPs, we dissociated secondary spheres expressing MYCN or a control construct and measured the number of tertiary spheres formed. The ratio of tertiary spheres formed per secondary sphere dissociated was significantly greater in the context of MYCN expression (Fig 3D). Therefore, not only does MYCN stimulate proliferation, but it also confers increased colony-forming potential.

### MYCN induces sympathoadrenal progenitor apoptosis

**Fig 4:**
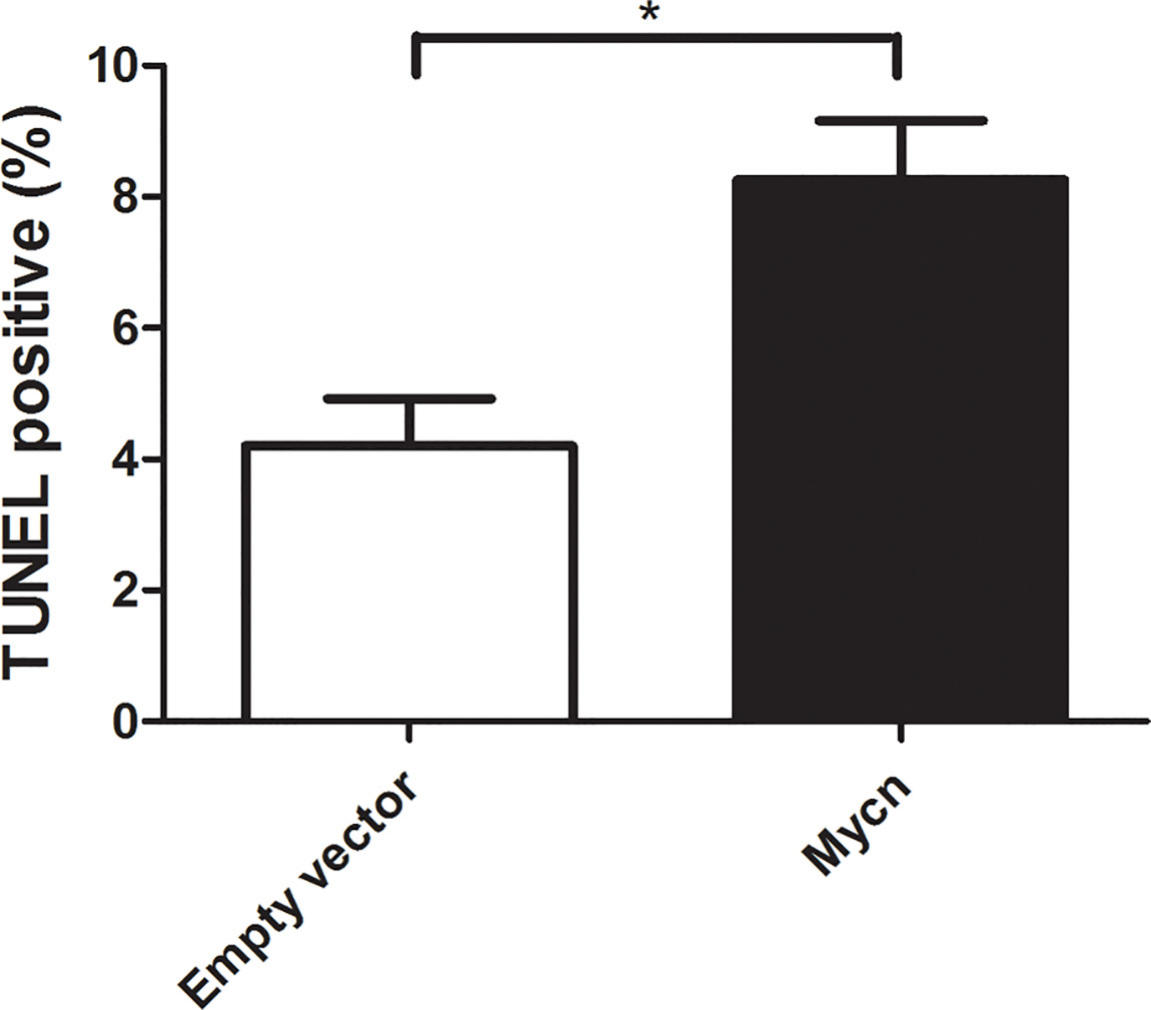
MYCN Induces SAP Apoptosis. Mycn induces apoptosis of sphere cells as evidenced by greater numbers of TUNEL-positive cells. Primary adrenal sphere cells were dissociated 4 days after infection with either empty or *Mycn* lentivirus. Secondary spheres were grown for 4 days and then dissociated, plated on coated dishes in self-renewal medium, and fixed and TUNEL stained following attachment (n = 3, p < .05, Student’s t-test).

Neuroblastoma tumors include a subset of dying cells, and measures of karyorrhexis correlate directly with tumor aggressiveness [39]. The recent finding that MYCN overexpression induces caspase-3 in zebrafish neuroblasts [17] raises the possibility that oncogene activity may be an important cause of cell death in SAPs and in tumor cells. To determine whether MYCN induces cell death in mammalian SAPs, we performed TUNEL labeling of cells from spheres infected with control or MYCN expressing lentivirus. MYCN overexpressing sphere cells showed more cell death, with approximately twice the rate of apoptosis (Fig 4).

### MYCN expression is not sufficient for tumor formation

**Fig 5:**
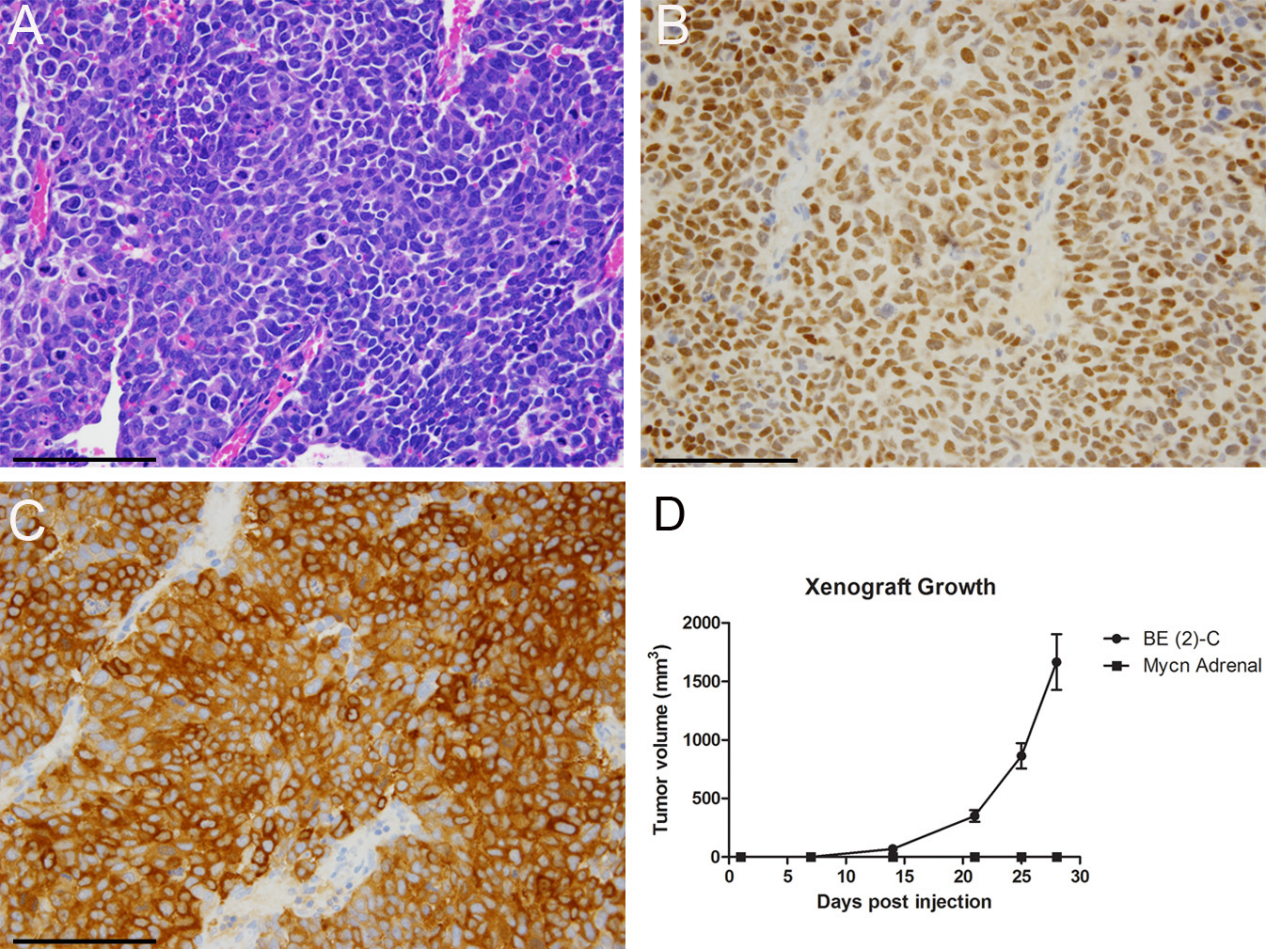
MYCN Expressing Progenitors Do Not Form Tumors. (A) Tumors derived from subcutaneously injected BE (2)-C cells showed neuroblastoma-like histologic features including dense cellularity, high nuclear-to-cytoplasmic ratios, high mitotic activity, and frequent apoptotic bodies. Scale bar equals 100 microns. (B) BE (2)-C derived tumors showed diffuse nuclear expression of PHOX2B and (C) diffuse cytoplasmic TH expression. Scale bars equal 100 microns. (D) While human neuroblastoma cells formed tumors in 6/7 mice, MYCN overexpressing adrenal progenitor cells did not form tumors (0/7). Average tumor volumes are shown. 5 x 10^4^ BE (2)-C human neuroblastoma cells or cells dissociated from *Mycn*-infected primary spheres were resuspended in HBSS with 30% Matrigel and delivered subcutaneously to nude mice.

Given that MYCN overexpression imparts tumor-like features including enhanced proliferation, neural lineage commitment, and colony forming potential, we assessed the ability of MYCN overexpressing adrenal progenitor cells to form tumors *in vivo*. Fifty thousand cells dissociated from primary spheres infected with MYCN expressing lentivirus were resuspended in HBSS with 30% matrigel and injected subcutaneously into the flanks of male nude mice. As a positive control, the same number of cells from the *MYCN*-amplified human neuroblastoma cell line BE (2)-C were delivered in an identical manner. While tumors with neuroblastoma-like histology and expression of the neuroblastoma SAP lineage markers Phox2B and TH developed in 6 out of 7 mice receiving BE (2)-C cells, no tumors formed following injection of the MYCN-overexpressing progenitor cells (Fig 5), indicating that overexpression of this oncogene in multipotent SAPs is not sufficient for tumorigenesis.

## Discussion

Following delamination from the dorsal neural tube, neural crest cells migrate widely and differentiate into a variety of derivatives. These derivatives include melanocytes of the skin, myelinating and non-myelinating Schwann cells, pericytes and smooth muscle cells of the vascular system, bone and cartilage tissue in portions of the face and teeth, and neurons of the dorsal root ganglia and autonomic (sympathetic and parasympathetic) ganglia (recently reviewed by La Noce et al. [40]). Sympathetic neurons form from a group of trunk neural crest cells that first differentiate into sympathoadrenal progenitors, which also give rise to adrenal medulla cells. The pediatric cancer neuroblastoma is thought to arise from these sympathoadrenal precursors, with tumors originating in the paraspinal sympathetic ganglia and the adrenal medulla [1,3,9,10]. Given that sympathoadrenal progenitor cells (SAPs) are the likely cells of origin for neuroblastoma, and that *MYCN* plays a key role in neuroblastoma development and prognosis, it is important to understand the effects of MYCN expression on SAPs. Few studies have directly addressed the influence of MYCN on non-neoplastic progenitor cells, and this is the first study to characterize the oncogene’s impact on primary mammalian sympathoadrenal progenitors.

We cultured SAPs as spheres from early postnatal mouse adrenal glands, and observed expression of NCSC and SAP markers in these cells. While spheres also expressed genes characteristic of sympathetic neuroblasts (e.g. tyrosine hydroxylase reactivity at the periphery), the NCSC/SAP profile and the capacity for secondary sphere formation with multi-lineage differentiation indicates a progenitor population. We found that overexpressing the murine *MYCN* homolog *Mycn* led to a dramatic increase in sphere size, with a significant increase in proliferation rate of sphere cells. MYCN expression also increased the proliferation rate of adrenal-derived adherent neurons in culture. These findings are compatible with previous *in vivo* studies showing increased numbers of neuroblasts in the MYCN transgenic zebrafish interrenal gland and TH-MYCN mouse sympathetic ganglia (hyperplasias) [17,41], and are consistent with a previous *in vitro* study showing MYCN-induced proliferation of chick sympathetic chain neurons *in vitro* [42].

Beyond its effect on proliferation, we also observed enhanced sphere-forming ability with MYCN overexpression, with greater numbers of tertiary spheres formed per secondary sphere dissociated. Enhanced sphere-forming ability is often used as an indicator of self-renewal [18,43,44]. In our system, MYCN simultaneously enhanced sphere-forming potential while promoting neural lineage commitment, and therefore does not promote “self-renewal” as strictly defined with maintenance of multipotency in subsequent colonies [21]. MYCN did, however, convey increased colony-forming potential in this system, raising the possibility that MYCN overexpression alone might permit SAPs to form a viable neoplasm, where pro-proliferative and survival factors outweigh factors promoting cell death. Compatible with this possibility are the findings that transgenic expression of the human *MYCN* gene in mouse neural crest cells induces neuroblastoma formation in sympathetic ganglia [14,15,41] while overexpression in zebrafish induces tumor formation in the adrenal gland equivalent [17].

We find, however, that MYCN overexpression alone in primary SAPs is not sufficient for tumor formation in nude mice. This finding stands in contrast to a similar experiment by Schulte and colleagues who found that overexpressing MYCN in neural crest progenitor cells was sufficient to permit formation of neuroblastoma-like tumors *in vivo* [16]. Schulte and colleagues used the neural crest progenitor cell line JoMa1, while we infected primary adrenal cells with the oncogene. While it is possible that MYCN overexpression alone may be sufficient for human tumor formation, we suggest that additional changes in gene expression, which likely characterize transgenic tumors and cell lines, are required for engraftment and tumor growth. Weiss et al, for example, found recurrent chromosomal gains and losses in MYCN transgenic mice, suggesting that genetic mutations in addition to misexpressed MYCN were required to promote neuroblast transformation [14,2]. The long delay between initiation of MYCN expression and tumor generation in that mouse model also supports the concept that additional genetic mutations are necessary [41,45]. Mutation of the ALK tyrosine kinase receptor occurs together with *MYCN* amplification in a subset of human neuroblastomas [46] and may be one such tumor-promoting mutation. Perhaps ALK-induced upregulation of TRKB [45], a protein shown to enhance the survival of neuroblastoma-derived cells [47], may act as a pro-survival factor in the context of MYCN-driven tumor development.

In tumors and developing tissues, MYCN expression inhibits neuronal maturation. The hyperplastic precursor lesions of transgenic MYCN mice include cells which express the neural progenitor marker Phox2B, but lack full neuronal maturation as indicated by absence of tyrosine hydroxylase expression [27]. Inhibition of MYCN expression *in vitro* results in maturation of neuroblastoma cell lines, with neurite outgrowth and upregulation of the differentiation markers neurofilament and GAP43 [28,29]. Similarly, many studies demonstrate reduced MYCN expression in cell lines induced to differentiate by retinoic acid exposure [48,49]. MYCN antagonizes differentiation in the central nervous system as well, where mice lacking the oncogene show precocious neural differentiation in the subventricular zone, as well as increased tubulin beta-3 expression and neuritic extensions [30]. We found that Mycn enhanced neural differentiation, as defined by neural lineage commitment, of multipotent SAPs with more neuron-containing colonies formed. A previous study of neural crest progenitors described a similar result: Wakamatsu and colleagues showed that MYCN overexpression induced early ventral migration of avian neural crest progenitors, with premature neuronal differentiation of cells in ganglion-forming areas [50]. A recent study found MYCN gene expression to be required for neuroblastoma cell line differentiation, with an increase in MYCN expression during early phases of retinoic acid-induced differentiation and a strong reduction of neurite formation with MYCN silencing [51]. Considered together with our findings, the results of these studies suggest that MYCN expression predisposes multipotent SAPs to a neural fate, while simultaneously limiting their potential for full neuronal maturation.

A neural-lineage promoting role for MYCN is very interesting in the context of the neuroblastoma histologic phenotype, where even poorly differentiated tumors show diffuse reactivity for the neural marker synaptophysin, and truly “undifferentiated” tumors are rare [39]. The MYCN target genes responsible for inducing neural differentiation have not been studied extensively. It has been shown that MYCN can bind to promoter and enhancer regions of the *ASCL1* (*MASH1*) gene [52], a pro-neural transcription factor required for sympathoadrenal neural differentiation [53]. And Yazawa et al. showed that MYCN can regulate ASCL1 expression [54]. Future studies will explore the downstream targets of MYCN in neural differentiation, and examine the requirement for ASCL1 in MYCN-induced neural lineage commitment.

In addition to its effects on proliferation and differentiation, we observed that MYCN expression increases the rate of apoptotic cell death in SAPs. This finding is compatible with those of Zhu et al, who found caspase-3 induction in interrenal gland neuroblasts of zebrafish overexpressing MYCN [17]. When the highly homologous oncogene *MYCC* is overexpressed *in vivo*, pancreatic beta-cell apoptosis occurs through an ARF-p53 dependent mechanism [55]. Interestingly, neuronal cells from the pre-cancerous sympathetic ganglia of TH-MYCN transgenic mice are resistant to apoptosis induced by nerve growth factor (NGF) withdrawal [41]. It has also been shown that silencing MYCN in neuroblastoma cell lines results in apoptotic cell death [56,57]. The influence of MYCN on survival may therefore depend on the degree to which a sympathoadrenal cell has progressed along the spectrum of tumorigenesis: in naïve primary cells MYCN would function as a gate-keeper, only permitting cells that have successfully down-regulated its expression to survive and progress further in differentiation. Pre-cancerous or cancerous cells would have evolved to require oncogene expression for maintenance of inappropriate survival pathways. The cellular response to MYCN may also depend on the level of oncogene expression as shown for MYCC by Murphy and colleagues, with a proliferative oncogenic response at lower levels and apoptosis at higher expression levels [55]. The *in vitro* model we describe is uniquely suited to explore the survival pathways that overcome MYCN’s pro-apoptotic effects, as well as to test the effects of oncogene expression level on cellular response.

## Conclusions

Here we have shown that MYCN imparts neuroblastoma tumor-like characteristics, including enhanced proliferation, neural lineage commitment, and higher rates of cell death, to multipotent sympathoadrenal progenitor cells. Hence, these findings support the hypothesis that multipotent sympathoadrenal progenitor cells are cells of origin for neuroblastoma. Further, we have presented an *in vitro* system for modeling the changes that may occur in the initial steps of neuroblastoma development. Future studies will examine the impact of MYCN expression on human sympathoadrenal progenitor cells, and explore the hypothesis that additional gene expression changes cooperate with MYCN to permit neuroblastoma growth.

## Supporting Information

S1 Fig Clonal Density Determination. (A) To determine clonal density parameters for primary sphere formation, single cells isolated from the adrenal gland by differential plating were grown in ultra-low attachment plates at 1, 2, or 4 cells per microliter of self-renewal medium. The number of resulting spheres was quantified after 6 days of growth. The number of spheres formed doubles between 1 and 2 cells per microliter plated; this indicates that cell clumping is an uncommon occurrence in this density range. Therefore 1 cell per microliter is a density at which each sphere formed typically arises from a single cell (clonal density). Experiments were performed 3 times. (B) To determine clonal density for secondary sphere formation, primary spheres were dissociated and plated at 1, 2, or 4 cells per microliter. The number of spheres formed roughly doubles between 1 and 2 cells per microliter plated, indicating that 1 cell per microliter is a density at which each sphere typically arises from a single cell. Experiments were performed 5 times. (C) To determine clonal density in the context of lentiviral infection, primary spheres infected with control lentivirus were dissociated and plated at 1, 2, or 4 cells per microliter in self-renewal medium in low adherence plates. The number of spheres formed doubles between 1 and 2 cells per microliter plated, indicating that 1 cell per microliter is a density at which each sphere typically arises from a single cell. Experiments were performed 4 to 6 times.
(TIF)

S1 Table Primer Sequences. (DOC)
