# Supplementary figures and images for: Expression of MYCN in Multipotent Sympathoadrenal Progenitors Induces Proliferation and Neural Differentiation, but Is Not Sufficient for Tumorigenesis
Source: PLoS One. 2015 Jul 29;10(7):e0133897. doi: 10.1371/journal.pone.0133897 (PMC4519318; doi:10.1371/journal.pone.0133897)

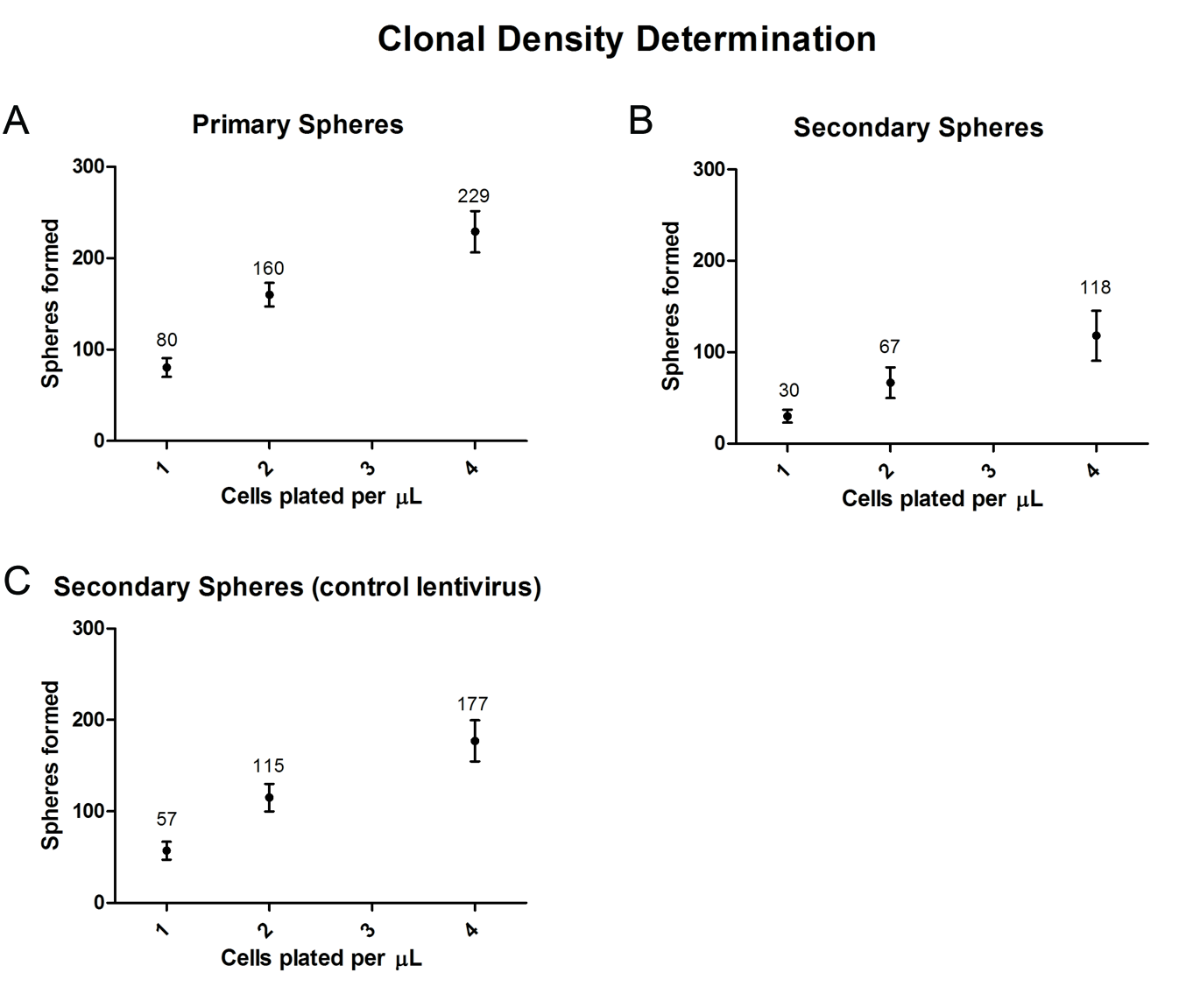

Supplement: S1 Fig — (A) To determine clonal density parameters for primary sphere formation, single cells isolated from the adrenal gland by differential plating were grown in ultra-low attachment plates at 1, 2, or 4 cells per microliter of self-renewal medium. The number of resulting spheres was quantified after 6 days of growth. The number of spheres formed doubles between 1 and 2 cells per microliter plated; this indicates that cell clumping is an uncommon occurrence in this density range. Therefore 1 cell per microliter is a density at which each sphere formed typically arises from a single cell (clonal density). Experiments were performed 3 times. (B) To determine clonal density for secondary sphere formation, primary spheres were dissociated and plated at 1, 2, or 4 cells per microliter. The number of spheres formed roughly doubles between 1 and 2 cells per microliter plated, indicating that 1 cell per microliter is a density at which each sphere typically arises from a single cell. Experiments were performed 5 times. (C) To determine clonal density in the context of lentiviral infection, primary spheres infected with control lentivirus were dissociated and plated at 1, 2, or 4 cells per microliter in self-renewal medium in low adherence plates. The number of spheres formed doubles between 1 and 2 cells per microliter plated, indicating that 1 cell per microliter is a density at which each sphere typically arises from a single cell. Experiments were performed 4 to 6 times. (TIF) [file pone.0133897.s001.tif]
