# Supplementary material for: Expression of MYCN in Multipotent Sympathoadrenal Progenitors Induces Proliferation and Neural Differentiation, but Is Not Sufficient for Tumorigenesis
Source: PLoS One. 2015 Jul 29;10(7):e0133897. doi: 10.1371/journal.pone.0133897 (PMC4519318; doi:10.1371/journal.pone.0133897)
Supplement: S1 Table — (DOC) [file pone.0133897.s002.doc]

| Supplementary Table 1. Primer sequences. | | |  |
| --- | --- | --- | --- |
|  | |  |  |
| **Gene/Accession number** | | **Primer sequences (F = forward, R = reverse)** | **Amplicon** |
|  | |  |  |
| Bmi1/NM_007552 | | **F** AAATCCCCACTTAATGTGTGTCC **R** CTTGCTGGTCTCCAAGTAACG | 118 |
| Mycn/NM_008709 | | **F** GCGGTAACCACTTTCACGAT **R** CTCTTCGCTTTTGCTGGAAC | 230 |
| Snai1/NM_011427 | | **F** CACACGCTGCCTTGTGTCT **R** GGTCAGCAAAAGCACGGTT | 133 |
| Sox10/NM_011437 | | **F** CTGCTGCTATTCAGGCTCACT **R** CGGACTGCAGCTCTGTCTTT | 156 |
| Nestin/NM_016701 | | **F** GATCGCTCAGATCCTGGAAG **R** TCAGGAAAGCCAAGAGAAGC | 153 |
| Ascl1/NM_008553 | | **F** TCTCCTGGGAATGGACTTTG **R** CCCCTGTTTGCTGAGAACAT | 188 |
| Phox2b/NM_008888 | | **F** TGAGACGCACTACCCTGACA **R** TCAGTGCTCTTGGCCTCTTT | 219 |
| Th/NM_009377 | | **F** TCTCCTTGAGGGGTACAAAACC **R** ACCTCGAAGCGCACAAAGT | 151 |
| Dbh/NM_138942 | | **F** TCCATCTGGATTCCCAGCAAG **R** ATGCAGGCCTGAGGTGTTGA | 199 |
| TrkA/NM_001033124 | | **F** GGCGATGACGTGTTTCTGC **R** AGGAGACGCTGACTTGGACA | 229 |
| Prph/NM_001163588 | | **F** GAACTGGAGCGCAAGATTGAG **R** CCGCGATGTTCTCGTACTGTG | 193 |
| Tbp1/NM_008948 | | **F** ACCCTGCCGTACCTTGTCT **R** TGTCTGTCGGGTAGAAGTTTTGA | 141 |
| Cyp11a1/NM_019779 | **F** GGATGCGTCGATACTCTTCTCA **R** GGACGATTCGGTCTTTCTTCCA | | 146 |
| Cyp11b2/NM_009991 | **F** CTGAACCGAAATGTGCTGTCA **R** CCTAGCCGTTCCCCAAAAAG | | 200 |
| SF-1/NM_139051 | **F** GAATGGCCGACCAGACCTTTA **R** GCAGCTCGCTCCAACAGTT | | 117 |
